# Revealing Physiological Basis for Floret Opening Difference Between Indica and Japonica Rice: Based on Floral Structure, Transcriptome, and Endogenous Floret Opening Regulator

**DOI:** 10.3390/genes15111396

**Published:** 2024-10-30

**Authors:** Ruyue Deng, Zhiqiang Yan, Huihui Tang, Susong Zhu

**Affiliations:** Guizhou Provincial Academy of Agricultural Sciences, Guiyang 550000, China; 17311992182@163.com (R.D.); tanghuihui0609@163.com (H.T.); 17880952854@163.com (S.Z.)

**Keywords:** floret opening, indica and japonica rice, floral organ, RNA-seq

## Abstract

Background: The differing floret opening times between subsp. *indica* and subsp. *japonica* in rice limit the potential for increased hybrid seed production. Objectives: To elucidate the physiological basis underlying the differences in floret opening time between indica and japonica rice. Materials: A comparative analysis involved nine indica and ten japonica rice varieties. Methods: Using paraffin sectioning, transcriptome sequencing, RT-PCR, and endogenous substance quantification, we investigated the structural variations in floral organs, the differences in the initiation timing of floret opening regulatory pathways, and endogenous regulators. Results: The results indicated insignificant differences in lemma thickness, lodicule thickness, lodicule area, and the coupling-lodicule length between indica and japonica rice. However, japonica rice exhibited larger lodicule-lemma gaps and more vascular bundles compared to indica rice. Within the 9:00 a.m. to 10:00 a.m. interval, the expression of *OsAOS1* in α-linolenic acid metabolism and *OsISA3* in starch and sucrose metabolism notably increased in indica rice, with no significant change in japonica rice. Additionally, the endogenous JA and α-amylase surged more significantly in indica rice than in japonica rice. The increase in soluble carbohydrate in indica rice is greater than in japonica rice, but the difference is not significant. Conclusions: These findings suggest that in the process of the floret opening, the α-linolenic acid metabolism and starch and sucrose metabolism are initiated earlier in indica rice, accompanied by a more pronounced elevation in endogenous JA and α-amylase. Furthermore, the smaller lodicule-lemma gap in indica rice contributes to earlier floret opening compared to japonica rice.

## 1. Introduction

The exploitation of heterosis between subsp. *indica* and subsp. *japonica* in rice holds significant potential for enhancing rice yield per unit area. However, the difference in floret opening time between these two subspecies poses a challenge to enhancing seed production yields [1,2]. Therefore, uncovering the intrinsic causes of floret opening time differences between subsp. *indica* and subsp. *japonica* in rice offers a vital theoretical basis for enhancing indica–japonica hybrid seed production yields.

Previous studies elucidated that floret opening is initiated by turgor movement of the lodicules [3], with floret opening time being a genetically determined trait [4,5,6]. The response patterns of floret opening to the environment, including temperature, humidity, CO_2_, light, and mechanical stimuli, have been unveiled [7,8,9,10,11,12]. Multiple QTLs regulating rice floret opening have been mapped to chromosomes such as chr1, chr2, chr6, chr7, chr8, chr10, and chr12 [13,14]. Numerous genes regulating floret opening have been cloned, including *EMF1*, *DFOT1*, *OsMYB8*, *OGI*, *OSNCED*, *OsPYL1*, *OsJAR1*, *OsAOS1*, *OsMYC2*, and *OsOPR7* [1,15,16,17,18,19,20,21]. Furthermore, various endogenous regulators of floret opening have been identified, such as endogenous jasmonic acid (JA), soluble carbohydrates, and K^+^ [3,22,23], alongside multiple metabolic pathways, including plant hormone signal transduction, starch and sucrose metabolism, and the α-linolenic acid pathway [24,25]. Moreover, exogenous chemicals modulating floret opening have been discovered, with methyl jasmonate (MeJA), epibrassinolide (epi-BR), and gibberellic acid (GA3) promoting floret opening [26,27,28,29], while methyl salicylate (MeSA), auxin (IAA), and naphthalene acetic acid (NAA) delay it [30,31,32]. Notably, IAA and NAA also facilitate the closure of florets. Subsp. *indica* in rice floret opening is earlier than subsp. *japonica* in rice, attributed to varying sensitivities to temperature, humidity, and grain shape [6,10]. However, few studies have examined structural differences in floral organs, especially lodicules, between the two subspecies.

Additionally, while multiple floret regulatory pathways are uncovered, their initial time in the process of the floret opening remains unexplored between the two subspecies. Hence, using 9 indica and 10 japonica rice varieties, this study examined the relationship between floret opening differences and floral organ structure. Transcriptome analyses at 9:00 a.m. and 10:00 a.m. on selected varieties aimed to uncover differences in floret opening regulatory pathway initiation, along with endogenous floret opening regulators, clarifying the physiological basis for earlier floret opening in indica rice.

## 2. Material and Methods

### 2.1. Planting Materials

Nine indica and ten japonica rice varieties (Table 1) were planted in the experimental field of the Rice Research Institute, Guizhou Provincial Academy of Agricultural Sciences, in 2024. Seeding was conducted on 13 April, and transplanting was performed on 21 May. Each variety was planted in four rows with ten plants per row, maintaining a plant-to-plant and row-to-row spacing of 15 × 30 cm.

### 2.2. Measurement of Floret Opening Time

Floret opening time was observed on the same day, and the observations were made over three sunny days. The floret opening time was defined as the moment when the first spikelet on an individual rice panicle reached full bloom, with this observation repeated three times. To ensure uniformity and standardization, the floret opening times were recalculated and expressed as the elapsed time from Beijing Time 8:00 a.m. to the precise moment of the first spikelet bloom [10]. The floret opening time data for all varieties and the corresponding weather information are provided in Table S1 and Table S2, respectively.

### 2.3. Observation of Floral Organ Anatomical Structures

Observations of floral organs were made on both transverse and longitudinal sections of the spikelets from nine indica and ten japonica rice varieties. The upper 1/2 of the unopened spikelet shell was first trimmed off, followed by immersion in a 70% FAA fixative for 24 h. Subsequently, the tissues were dehydrated and cleared using a series of graded ethanol and xylene solutions, respectively. These tissues then underwent infiltration and embedding with paraffin, sectioning (8 μm thickness), transverse section slightly below the central part of lodicule, dewaxing, rehydration, and staining with 1% safranin and 0.5% fast green [33]. The prepared slides were scanned using a 3D HISTECH (Pannoramic Scan; Pannoramic, Hungary) scanner, and measurements with CaseViewer software 2.3. The measurements of the floral organs are detailed in Table S3.

### 2.4. RNA Extraction and Sequencing

Spikelets of indica rice HFSM and japonica rice JD104 were sampled at 9:00 a.m. and 10:00 a.m. under the same sunny day for a transcriptome analysis, ensuring triplicate biological replication for each time. RNA extraction was accomplished utilizing the Trizol reagent (Invitrogen, Waltham, MA, USA). After quality and purity analyses using Bioanalyzer 2100 and the RNA 6000 Nano LabChip Kit (Agilent, Santa Clara, CA, USA, 5067–1511), only samples with an RIN > 7 were deemed suitable for the construction of sequencing. Following extraction, the RNA underwent purification using Dynabeads Oligo (dT) (Thermo Fisher, Waltham, MA, USA). The mRNA was cleaved into shorter fragments, which were then reverse-transcribed into cDNA by SuperScript™ II Reverse Transcriptase (Invitrogen, cat.1896649, Waltham, MA, USA). Through end repair and polyA addition and using DNA Sample Prep Master Mix Set 1 (New England Biolabs, Ipswich, MA, USA), sequencing adapters were added to the cDNA termini. Subsequently, agarose gel electrophoresis was performed for fragment size selection and purification, followed by PCR amplification. The 2 × 150 base pair (bp) paired-end sequencing (PE150) was carried out utilizing the Illumina Novaseq™ 6000 platform (LC-Bio Technology CO., Ltd., Hangzhou, China), adhering to the manufacturer’s guidelines. The Ensembl_v54 reference genome was utilized for the mapping using HiSAT2 [34]. To ensure high-quality clean reads, the reads were subsequently filtered using Cutadapt [35]. The experiment resulted in the construction of a total of 12 cDNA libraries (Table S4).

### 2.5. Quantitative RT-PCR Analysis

Spikelets of indica rice HFSM and japonica rice JD104 were sampled at 9:00 a.m. and 10:00 a.m. under the same sunny day for RT-PCR, aligning with the sampling times used for transcriptome sequencing. Following RNA extraction with an RNAiso Plus Reagent (Takara, Dalian, China), reverse transcription was conducted using SYBR Premix Ex Taq II, in adherence with the manufacturer’s instructions (Takara, Dalian, China). Primer information is in Table S5. The conditions for the reaction and the computational formulas employed were derived from previous studies [24].

### 2.6. Measurement of Endogenous JA, α-Amylase, and Soluble Carbohydrates

Endogenous JA was quantified using high-performance liquid chromatography (HPLC, Agilent 1290) and tandem mass spectrometry (Applied Biosystems 6500 Quadrupole Trap). The chromatographic conditions were as follows: chromatographic column—Agilent Poroshell 120 SB-C18 (2.7 μm, 2.1 × 150 mm); column temperature—40 °C; mobile phase A—0.1% formic acid in methanol; mobile phase B—0.1% formic acid in water; injection volume—2 µL; flow rate—0.2 mL/min; and elution profile—0–13.5 min. Mass spectrometer conditions were as follows: the nebulization temperature of electron spray ionization (ESI)—400 °C; the pressure for the curtain gas—15 psi; and gas pressure for the nebulizer—65 psi [36].

α-Amylase activity was determined using an α-amylase activity assay kit (Solarbio, 50T/24S, Beijing, China). Accurately weigh 0.1 g of the sample and add 0.8 mL of distilled water. Homogenize the mixture and allow it to stand for 15 min. Centrifuge at 6000 r/min for 10 min at room temperature. Collect the supernatant and adjust the final volume to 10 mL. Add 250 µL of Reagent B to the starch solution, and incubate the mixture in a 70 °C water bath for 15 min. After cooling to room temperature, add 500 µL of Reagent A and incubate the mixture in a 40 °C water bath for 5 min. Finally, mix thoroughly and incubate in a boiling water bath for 10 min. Measure the absorbance at 540 nm using a spectrophotometer (SpectraMax190, Molecular Devices, Sunnyvale, CA, USA) [37].

Soluble carbohydrate was quantified by anthrone colorimetry. A mixture was created by adding 0.3 g of spikelets of powder to 10 mL of distilled water. Following a 30 min boiling period, the supernatant was retrieved. Next, we repeated the previous step until the collected supernatant volume reached 25 mL. Finally, measurements were conducted using an anthrone-ethyl acetate reagent and concentrated sulfuric acid [37]. The experiments mentioned were carried out with three repetitions.

### 2.7. Statistical Analyses

The statistical analysis was performed using SPSS 20.0 for *t*-tests, while GraphPad Prism 9 was utilized for the creation of visual representations. The assembly and enhancement of images were conducted in Photoshop.

## 3. Results

### 3.1. Investigation of Floret Opening Time of Both Indica and Japonica Rice

**Figure 1:**
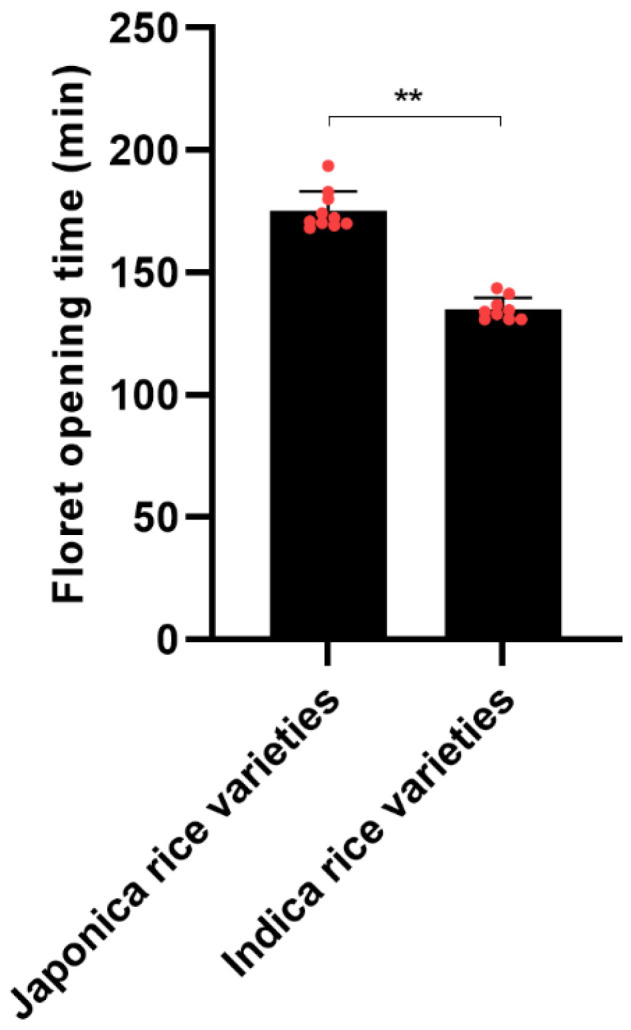
Investigation of floret opening time of both indica and japonica rice, using Student’s *t*-tests. “**” mean extremely significant at levels of 0.01. Each red dot represents a rice variety.

Preliminary research in Liaoning Province, Northeast China, has established that the floret opening of indica rice precedes that of japonica rice [27]. Affirming results from Guizhou Province, Southwest China, highlight a markedly earlier floret opening for indica rice (Figure 1). Consistent findings indicate a universal difference in the floret opening time of indica and japonica rice across diverse ecological regions.

### 3.2. Structural Differences in the Floral Organs of Indica and Japonica Rice

**Figure 2:**
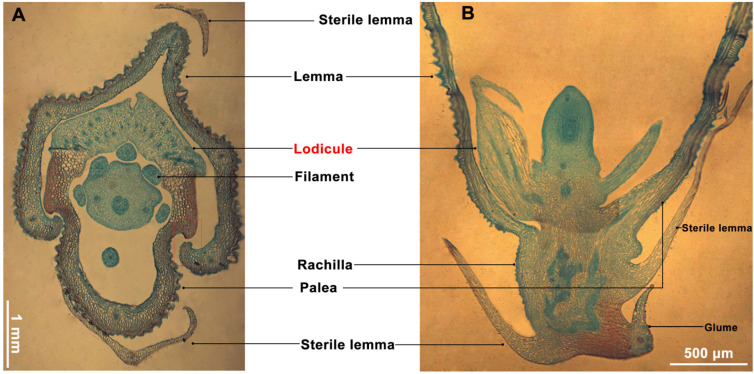
Structure of floret. (**A**) Transverse structure of floret. (**B**) Longitudinal structure of floret.

**Figure 3:**
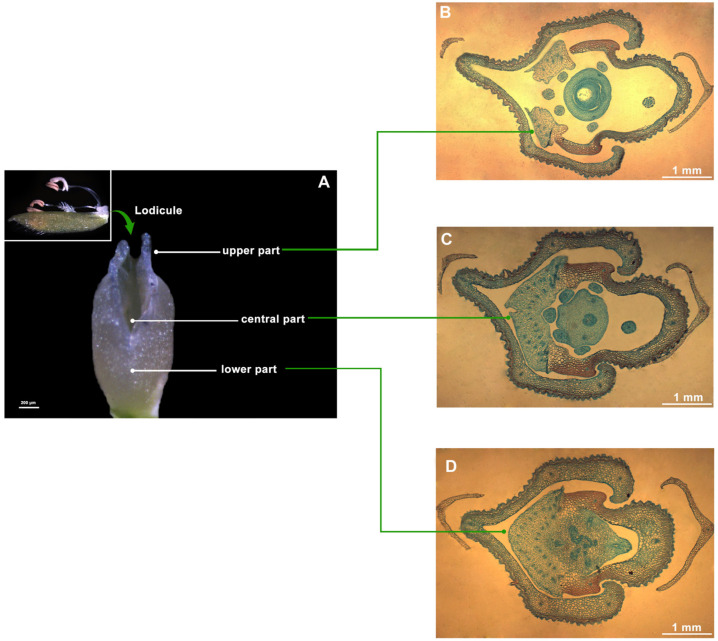
Lodicule structure observation at different positions. (**A**) The phenotype of the lodicule. (**B**–**D**) Structures of the upper, central, and lower parts of the lodicule.

The rice floret consists of the lemma, palea, stamens, pistil, lodicules, sterile lemma, and glume [21]. Rice floret opening is driven by turgor movement in the lodicules, which swell by absorbing water, forcing open the groove between the lemma and palea and applying pressure to the lemma, leading to floret opening (Figure 2A,B). The lower part of the lodicules is fully connected, the central part is partially connected, and the upper part is completely separate (Figure 3A). The vascular bundle distribution is more abundant in the lower and central part and less frequent in the upper part of the lodicules (Figure 3B–D).

**Figure 4:**
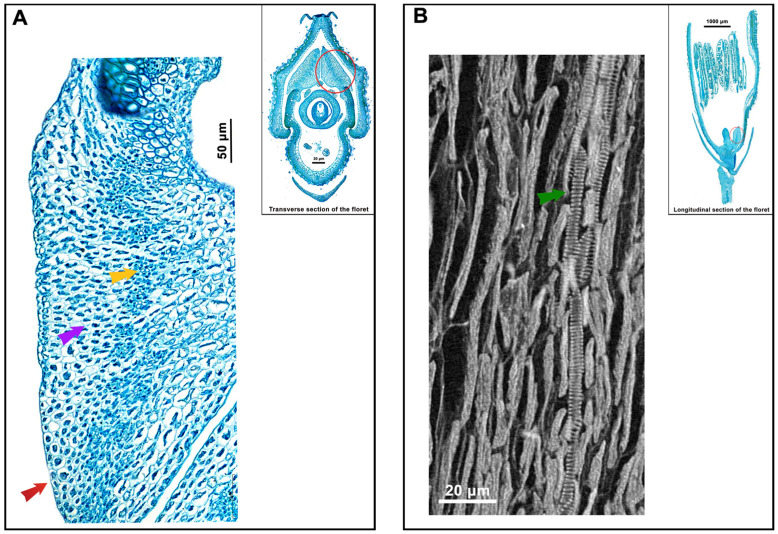
Lodicule cell structure. (**A**) Composition of lodicule cells. (**B**) Observation of vessel in lodicule. Red circle indicates area to be magnified. Red, purple, yellow, and green arrows indicate small parenchyma cells, large parenchyma cells, vascular bundles, and annular vessels, respectively.

**Figure 5:**
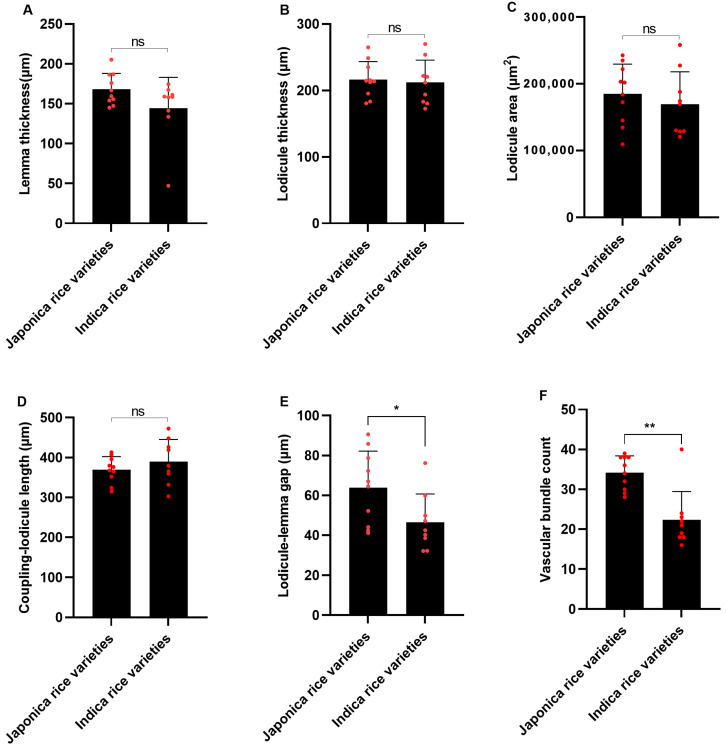
Morphological differences in floral organs between indica and japonica rice. (**A**) Glume thickness. (**B**) Lodicule thickness. (**C**) Lodicule area. (**D**) Coupling-lodicule length. (**E**) Lodicule-lemma gap. (**F**) Vascular bundle count. Student’s *t*-tests were used. “*” and “**” mean significant and extremely significant at the levels of 0.05 and 0.01, respectively. “ns” stands for no significant difference. Each red dot represents a rice variety.

The lodicules of both indica and japonica rice consist of an outer layer of small parenchyma cells, an intermediate layer of large parenchyma cells, and vascular bundles. The cytoplasm of the parenchyma cells is denser. The vascular bundles lack pronounced bundle sheath cells and there is no distinct separation into the xylem and phloem. Each vascular bundle contains an indeterminate number of vessels (Figure 4A), all of which are annular vessels (Figure 4B). The comparative analysis reveals no significant variance in the lemma thickness, cross-sectional lodicule thickness and area, and coupling-lodicule length between indica and japonica rice (Figure 5A–D). However, japonica rice features a pronouncedly wider lodicule-lemma gap, along with a greater number of vascular bundles, compared to indica rice (Figure 5E,F). In a previous study conducted in Liaoning, Northeast China, the authors observed differences in the number of vascular bundles in the lodicules among various indica and japonica rice varieties, which are consistent with the results of the current study (Figure S1). The results indicate that japonica rice has more developed vascular tissue, while the smaller lodicule-lemma gap in indica rice is more favorable for the lodicules to exert pressure on the lemma.

### 3.3. KEGG Annotation of Shared and Unique Significantly Differentially Expressed Genes (DEGs) Between HFSM_10_VS_9 and JD104_10_VS_9

**Figure 6:**
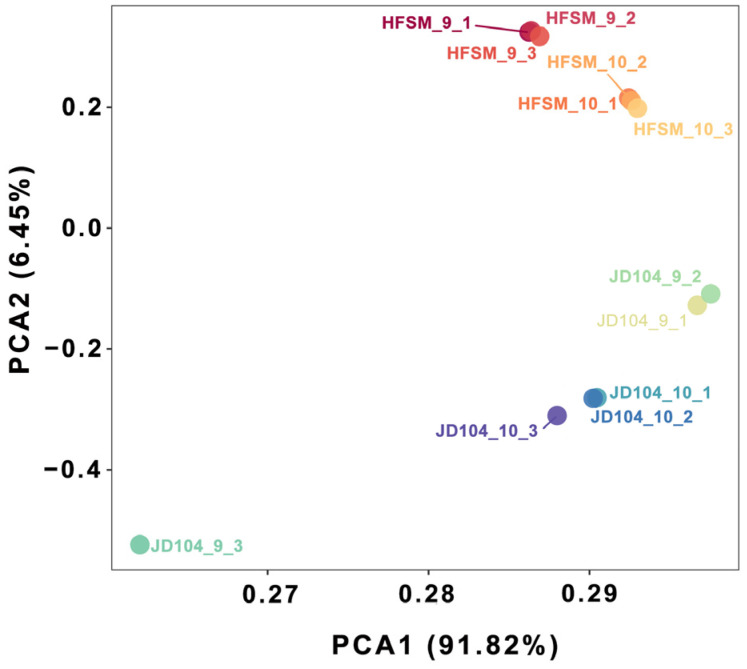
PCA plot of RNA-seq samples.

**Figure 7:**
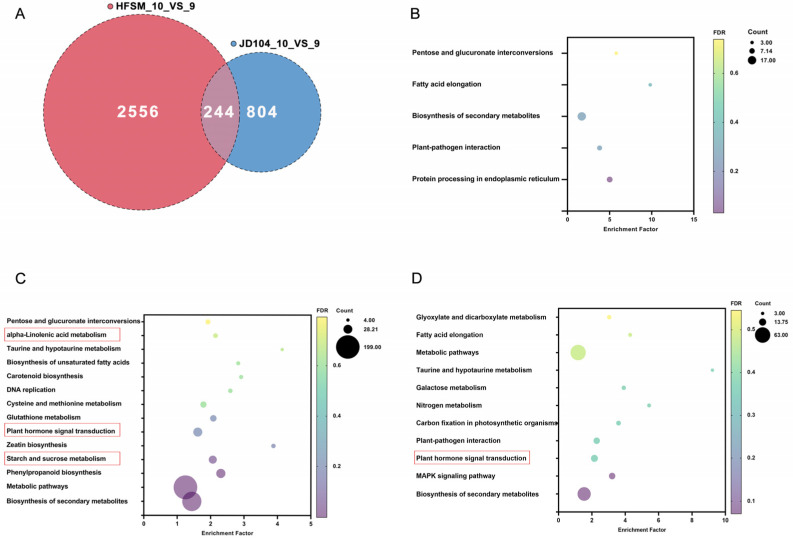
The KEGG annotation of significant DEGs between HFSM_10_VS_9 and JD104_10_VS_9. (**A**) A Venn diagram of the two comparison groups. (**B**) The KEGG annotation of the shared significant DEGs between the two comparison groups. (**C**) The KEGG annotation of the significant DEGs unique to the HFSM_10_VS_9. (**D**) The KEGG annotation of the significant DEGs unique to the JD104_10_VS_9. HFSM_10_VS_9 and JD104_10_VS_9 represent the comparative transcriptome analyses of HFSM and JD104 florets at 10:00 a.m. versus 9:00 a.m., respectively.

The PCA results indicated that all samples clustered well except for JD04_9_3 (Figure 6). There are 244 significant DEGs shared between HFSM_10_VS_9 and JD104_10_VS_9, and the numbers of significant DEGs unique to HFSM_10_VS_9 and JD104_10_VS_9 are 2556 and 804, respectively (Figure 7A). KEGG annotation shows that the shared significant DEGs are primarily involved in five pathways, including pentose and glucuronate interconversions, fatty acid elongation, biosynthesis of secondary metabolites, plant–pathogen interaction, and protein processing in the endoplasmic reticulum (Figure 7B). For HFSM_10_VS_9, the unique significant DEGs to HFSM_10_VS_9 are associated with 14 pathways, including pentose and glucuronate interconversions, α-linolenic metabolism, taurine and hypotaurine metabolism, biosynthesis of unsaturated fatty acids, carotenoid biosynthesis, DNA replication, cysteine and methionine metabolism, glutathione metabolism, plant hormone signal transduction, zeatin biosynthesis, starch and sucrose metabolism, phenylpropanoid biosynthesis, metabolic pathways, and biosynthesis of secondary metabolites (Figure 7C). JD104_10_VS_9 exhibits unique significant DEGs linked to 11 pathways, including biosynthesis of secondary metabolites, the MAPK signaling pathway, plant hormone signal transduction, plant–pathogen interaction, carbon fixation in photosynthetic organisms, nitrogen metabolism, galactose metabolism, taurine and hypotaurine metabolism, metabolic pathways, fatty acid elongation, and glyoxylate and dicarboxylate metabolism (Figure 7D).

### 3.4. Heatmap of Significant DEGs Specific to HFSM_10_VS_9 and JD104_10_VS_9

**Figure 8:**
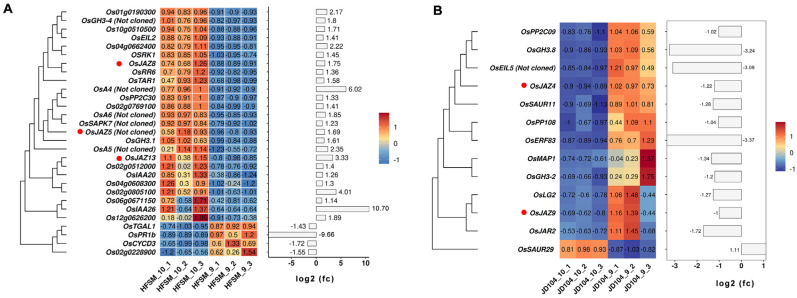
Heatmap analysis. (**A**,**B**) Heatmap of significant DEGs in plant hormone signal transduction unique to HFSM_10_VS_9 and JD104_10_VS_9, respectively. HFSM_10 and JD104_10 represent samples of HFSM and JD104 collected at 10:00 a.m., while HFSM_9 and JD104_9 are from 9:00 a.m. Subsequent numbers indicate biological replicates.

Previous studies showed that α-linolenic metabolism, plant hormone signal transduction, and starch and sucrose metabolism are important regulatory pathways involved in floret opening of rice [24]. Given that the specific regulatory pathways for HFSM_10_VS_9 and JD104_10_VS_9 both include plant hormone signal transduction, which includes the *JAZ* genes participating in rice floret opening (Figure 8A,B), this study focuses primarily on the analysis of genes in two additional pathways unique to HFSM_10_VS_9 beyond plant hormone signal transduction.

**Figure 9:**
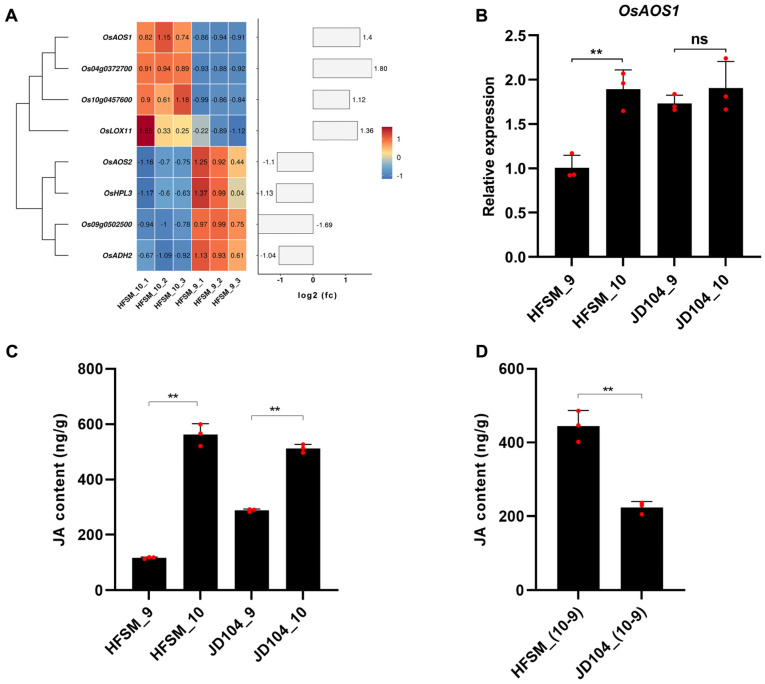
Analysis of α-linolenic metabolism pathway. (**A**) Heatmap of significant DEGs unique to HFSM_10_VS_9 in α-linolenic metabolism pathway. (**B**) Detection of *OsAOS1* expression levels by RT-PCR. (**C**) Comparative analysis of endogenous JA levels between HFSM and JD104 at 9:00 a.m. and 10:00 a.m., respectively. (**D**) Comparison of endogenous JA elevation between HFSM and JD104 during one-hour interval from 9:00 to 10:00 a.m. Student’s *t*-tests were used. “**” mean extremely significant at levels of 0.01. “ns” stands for no significant difference. HFSM_10 and JD104_10 represent samples of HFSM and JD104 collected at 10:00 a.m., while HFSM_9 and JD104_9 are from 9:00 a.m. Subsequent numbers indicate biological replicates. Each red dot represents one biological replicate.

Endogenous JA contributes to floret opening, with *OsAOS1* in α-linolenic metabolism being a crucial gene in JA biosynthesis. Mutations in *OsAOS1* result in delayed and scattered floret opening in rice [38], and its expression peaks close to floret opening [22,24]. In this study, the expression of *OsAOS1* in the α-linolenic metabolism pathway was notably increased in HFSM_10_VS_9 (Figure 9A). RT-PCR also confirmed that the expression of *OsAOS1* was significantly upregulated in HFSM_10_VS_9, with no significant upregulation observed in JD104_10_VS_9 (Figure 9B). A subsequent analysis of the endogenous JA confirmed a significant increase in JA content in both HFSM_10_VS_9 and JD104_10_VS_9 (Figure 9C). Notably, the upregulation of JA in HFSM_10_VS_9 was considerably more pronounced than in JD104_10_VS_9 (Figure 9D).

**Figure 10:**
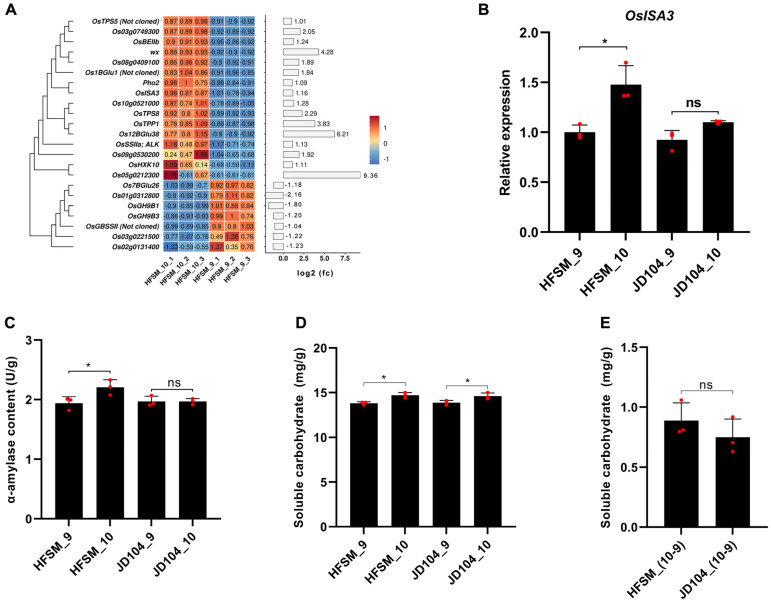
Analysis of starch and sucrose metabolism. (**A**) Heatmap of significant DEGs unique to HFSM_10_VS_9 in starch and sucrose metabolism. (**B**) Detection of *OsIsA3* expression levels by RT-PCR. (**C**,**D**) Comparative analysis of α-amylase and soluble carbohydrates between HFSM and JD104 at 9:00 a.m. and 10:00 a.m., respectively. (**E**) Comparison of soluble carbohydrate elevation between HFSM and JD104 during one-hour interval from 9:00 to 10:00 a.m. Student’s *t*-tests were used. “*”mean significant at levels of 0.05. “ns” stands for no significant difference. HFSM_10 and JD104_10 represent samples of HFSM and JD104 collected at 10:00 a.m., while HFSM_9 and JD104_9 are from 9:00 a.m. Subsequent numbers indicate biological replicates. Each red dot represents one biological replicate.

Soluble carbohydrates are primary osmotic regulators for lodicules. *OsISA3* in the starch and sucrose metabolism pathway can accelerate starch degradation and increase sugar supply to the grain [39]. In this study, transcriptome and RT-PCR analyses both indicate a significant upregulation of *OsISA3* expression in HFSM_10_VS_9, with no significant change observed in JD104_10_VS_9 (Figure 10A,B). A further analysis showed that the activity of α-amylase was significantly increased in HFSM_10_VS_9, while no significant change was observed in JD104_10_VS_9 (Figure 10C). Interestingly, the content of soluble carbohydrates significantly increased in both HFSM_10_VS_9 and JD104_10_VS_9 (Figure 10D), with no significant difference in the extent of the increase between the two (Figure 10E).

## 4. Discussion

### 4.1. Comparison of Vascular Tissue in Lodicule Between Indica and Japonica Rice

**Figure 11:**
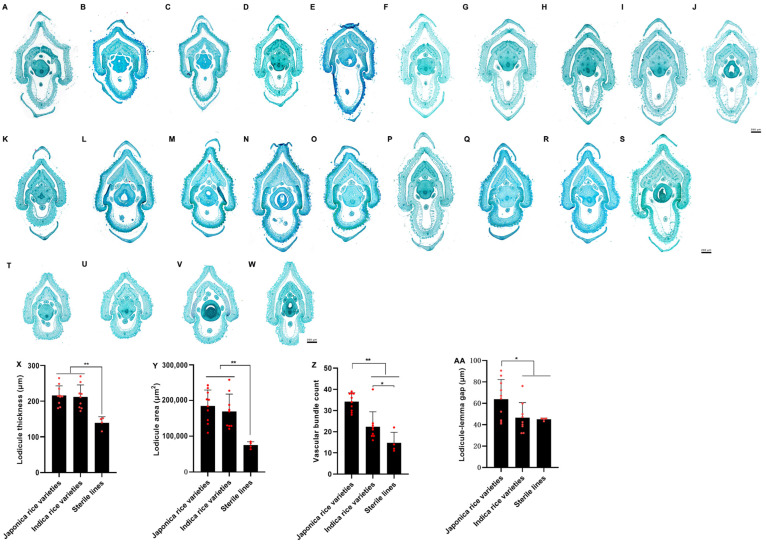
Analysis of floral organ morphology in indica, japonica, and sterile rice lines. (**A**–**J**) Japonica rice varieties: JD104, SN9816, JXG, HG1, JG88, JG81, CG7, BD08, YG22, JYE28. (**K**–**S**) Indica rice varieties: EFSM, CH727, GH963, QXH875, XYXZ, JMZ, CFB, IR8, HFSM. (**T**–**W**) Sterile lines: ShanxiangA, SixiangA, TaifengA, Shun6S. (**X**–**AA**) Statistical analysis of lodicule thickness, lodicule area, vascular bundle count, and lodicule-lemma gap in indica, japonica, and sterile rice lines, respectively. “*” and “**” mean significant and extremely significant at levels of 0.05 and 0.01, respectively. Each red dot represents a rice variety.

Previous studies indicated that the lack of vascular bundles and the vessels in the lodicule of sterile lines is the main reason for their delayed floret opening [40]. This study compared the floral organ structures of four sterile lines with those of the indica and japonica rice varieties used in this research. It was visually apparent that the lodicule of indica and japonica rice was more robust than that of the sterile lines (Figure 11A–W). A further analysis revealed that the lodicule thickness and area, as well as the vascular bundle count, were significantly greater in indica and japonica rice than in sterile lines (Figure 11X–Z). The lodicule-lemma gap of japonica rice was significantly greater than that of indica rice and sterile lines, whereas no significant difference was found between indica and sterile lines (Figure 11AA). The results above indicate that the poor floret opening habits in sterile lines are closely related to underdeveloped lodicules. Although indica rice has earlier floret opening time than japonica rice, the number of vascular bundles in indica rice is significantly lower than that in japonica rice (Figure 5F and Figure S1). This suggests that well-developed lodicules and narrower lodicule-lemma gaps are structural features that facilitate floret opening in both indica and japonica rice; advantageous floral organ structures are not the primary factor determining the time of floret opening between these subspecies. Instead, the timing of the initiation of endogenous floret opening regulatory pathways may be the decisive factor.

### 4.2. Analysis of Plant Hormone Signal Transduction and Starch and Sucrose Metabolism

Previous studies showed that the expression of *OsJAZ1*–*OsJAZ13* in plant hormone signal transduction, which is involved in the JA signaling pathway, is notably upregulated close to floret opening in rice [22,24]. The transcriptome analysis in this study indicates that during the one-hour interval from 9:00 a.m. to 10:00 a.m., the expressions of *OsJAZ5*, *OsJAZ8*, and *OsJAZ13* were significantly upregulated in HFSM (Figure 8A), while no significant changes were observed in JD104. The expression of *OsJAZ4* and *OsJAZ9* were notably downregulated in JD104 (Figure 8B), with no significant differences observed in HFSM. Given that JA promotes floret opening in rice and facilitates the degradation of *OsJAZ9*, and that knocking out the *OsJAZ9* enhances floret opening in rice [38], we speculate that the JA signaling pathway may regulate floret opening in indica and japonica rice through distinct mechanisms.

From 9:00 a.m. to 10:00 a.m., while the significant DEGs associated with starch and sucrose metabolism were enriched only in HFSM and α-amylase was significantly increased only in HFSM_10_VS_9, there was a significant increase in soluble carbohydrate content in both HFSM and JD104, with no significant difference observed between the two rice varieties (Figure 10D,E). Soluble carbohydrates are the main osmotic agents that drive the swelling of the lodicules due to hydration [31]. Prior research has shown that the soluble sugars of the lodicule originate not only from the hydrolysis of starch in the lodicule but also from contributions along the marginal tissues of the palea and rachilla [3,41]. Additionally, the research demonstrates that, in contrast to indica rice, japonica rice exhibits a more robust vascular tissue in the lodicule. This implies that the increase in soluble carbohydrate content in JD104 may be due to external translocation.

### 4.3. Regulatory Mechanism of Floret Opening Time Difference Between Indica and Japonica Rice

*OsAOS1*, a key gene in JA biosynthesis, regulates rice floret opening. The mutations in *OsAOS1* result in delayed and scattered floret opening in rice, and its expression peaks close to floret opening. The knockout mutation of the *OsAOS1* leads to a significant decrease in jasmonic acid (JA) within the lodicule; however, the exogenous application of methyl jasmonate (MeJA) can restore the phenotype [38]. This study revealed distinct haplotypes of *OsAOS1* in indica (Hap–1 and Hap–2) and japonica (predominantly Hap–1) rice (Figure S2), suggesting potential differential activity. This may lead to variations in *OsAOS1* expression and endogenous JA levels, consequently affecting floret opening between indica and japonica rice.

Previous research established that the *OsMYC2*-JA feedback loop modulates floret opening in rice. Specifically, *OsMYC2*, as a transcriptional regulator of *OsAOS1*, directly promotes *OsAOS1* expression, thereby enhancing endogenous JA and accelerating rice floret opening. Furthermore, the overexpression of *OsMYC2* results in earlier floret opening. Our study found that during the one-hour interval from 9:00 a.m. to 10:00 a.m., there were no significant changes in *OsMYC2* expression in both HFSM and JD104 (Figure S3). However, *OsAOS1* expression significantly increased in HFSM but remained stable in JD104, with a notably higher elevation of endogenous JA content in HFSM compared to JD104. The haplotype analysis indicated that *OsMYC2* exhibits distinct haplotypes in indica (predominantly Hap–2) and japonica (primarily Hap–1) rice (Figure S4). We speculate that these haplotype differences may confer varying activity, leading to differential *OsAOS1* expression between indica and japonica rice.

Our previous study showed that exogenous MeJA significantly promotes floret opening in both indica and japonica rice. However, applying MeJA did not synchronize their floret opening, with the indica rice floret opening earlier than japonica rice [27]. One hour after MeJA application, the expressions of JA signaling pathway genes in indica rice, such as *OsJAZ1*, *OsJAZ5*, *OsJAZ6*, *OsJAZ9*, *OsJAZ10*, *OsJAZ11*, and *OsJAZ12*, were substantially higher than in japonica rice. Genes in the α-linolenic acid signaling pathway (*OsAOC*, *OsAOS1*, *OsAOS3*, *OsAOS4*) and the starch sucrose metabolic pathway (*OsUPG2*, *Pho2*, *OsISA3*, *OsAGPL4*, *DPE1*, *GIF1*, *WX*, and *OsHXK10*) also exhibited significant expression changes in indica rice, with less pronounced changes in japonica rice. Additionally, the endogenous JA, MeJA, and α-amylase, which are key regulators of floret opening, were significantly higher in indica rice [2]. This suggests that inherent differences in the endogenous regulators or pathways involved in the floret opening may entrench the difference in the floret opening time between indica and japonica rice. Although MeJA can stimulate the regulatory pathways or substances associated with floret opening, it fails to eliminate the difference in floret opening between indica and japonica rice. In this study, despite a limited number of samples in the physiological experiments, previous research partially supports the results presented here.

## 5. Conclusions

**Figure 12:**
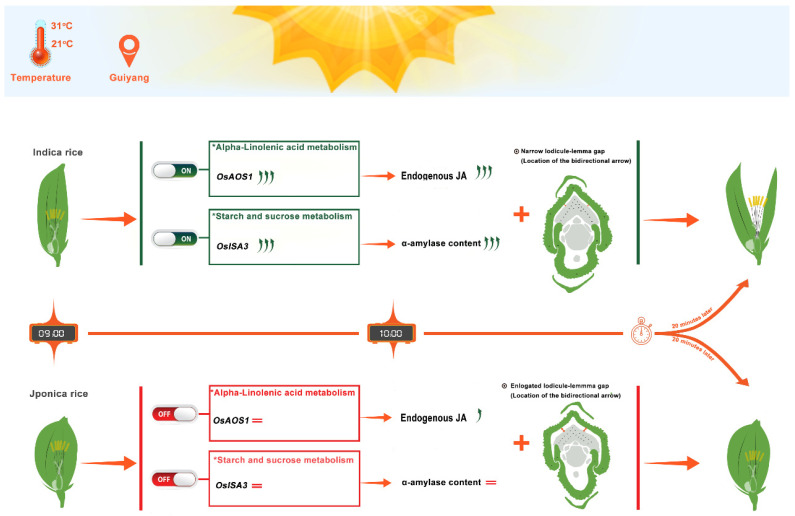
Physiological mechanism model of inconsistent floret opening time between indica and japonica rice. Green arrows: upregulation of gene expression or endogenous substance abundance, with arrow count indicating intensity. Red double lines: no significant change.

Indica rice exhibits an advanced initiation in α-linolenic acid metabolism and starch and sucrose metabolism, which correlates with a more significant increase in endogenous JA and α-amylase content in indica rice compared to japonica rice. This, along with the shorter lodicule-lemma gap characteristic of indica rice, contributes to its earlier floret opening (Figure 12).

## Figures and Tables

**Table 1:** Rice varieties’ information.

Cultivar Name	Variety Name Abbreviation	Subsp. *Indica*/ Subsp. *Japonica*	Heading Date
Efengsimiao	EFSM	Subsp. *Indica*	5 August 2024
Chenghui727	CH727	Subsp. *Indica*	1 August 2024
Guihui963	GH963	Subsp. *Indica*	1 August 2024
Qianxianghui875	QXH875	Subsp. *Indica*	1 August 2024
Xiangyaxiangzhan	XYXZ	Subsp. *Indica*	22 July 2024
Jinmazhan	JMZ	Subsp. *Indica*	22 July 2024
ChengfengB	CFB	Subsp. *Indica*	6 August 2024
IR8	IR8	Subsp. *Indica*	1 August 2024
Hengfengsimiao	HFSM	Subsp. *Indica*	22 July 2024
Jindao104	JD104	Subsp. *Japonica*	5 August 2024
Shennong9816	SN9816	Subsp. *Japonica*	1 August 2024
Jinxianggeng	JXG	Subsp. *Japonica*	25 July 2024
Hugeng1	HG1	Subsp. *Japonica*	22 July 2024
Jigeng88	JG88	Subsp. *Japonica*	28 July 2024
Jigeng81	JG81	Subsp. *Japonica*	5 August 2024
Chugeng7	CG7	Subsp. *Japonica*	1 August 2024
Bidao08	BD08	Subsp. *Japonica*	28 July 2024
Yonugeng22	YG22	Subsp. *Japonica*	28 July 2024
JinyuanE28	JYE28	Subsp. *Japonica*	28 July 2024

## Data Availability

Sequence data that support the findings of this study were deposited in the Genome Sequence Archive in the National Genomics Data Center, China National Center for Bioinformation/Beijing Institute of Genomics, Chinese Academy of Sciences (GSA), under accession number CRA018732.

## Supplementary Materials

The following supporting information can be downloaded at: https://www.mdpi.com/article/10.3390/genes15111396/s1, Figure S1. Observation of vascular bundles of the lodicule in indica and japonica rice. (A–D) Japonica rice varieties, respectively: Fengjing, Kongyu131, Akitakomachi, Koshihikari. (E–H) Indicate rice varieties, respectively: Zhaiyeqing8, Gang46B, Minghui100, Minghui70. (I) Comparison of vascular bundle count of lodicule between indica and japonica rice varieties. The blue and red dashed lines show the average vascular bundle numbers in japonica and indica rice lodicules. “**” mean extremely significant at the levels of 0.01. Figure S2. Haplotype analysis of *OsAOS1*. Figure S3. Detection of *OsMYC2* expression by RT-PCR. Figure S4. Haplotype analysis of *OsMYC2*. Table S1. Investigation of floret opening time. Table S2. Weather records. Table S3. The measurements of the floral organs. Table S4. RNA integrity number (RIN) and sequencing data. Table S5. Primer’s information.

## References

[1] Zhu X., Wang M., Huang Z., Chen M., Xu P., Liao S., Gao Y., Zhao Y., Chen H., He J. (2024). The OsMYC2–JA feedback loop regulates diurnal flower-opening time via cell wall loosening in rice. Plant J..

[2] Yan Z., Deng R., Tang H., Zhang H., Zhu S. (2024). Molecular basis of differential sensitivity to MeJA in floret opening between indica and japonica rice. Czech J. Genet. Plant Breed..

[3] Zeng X., Zhou X., Wu X. (2004). Advances in study of opening mechanism in rice florets. Sci. Agric. Sin..

[4] Hu N., Cao M., Jiang X., Hu J., Yao K. (2013). Estimating model for rice flowering habit with different rice varieties. Chin. J. Agrometeorol..

[5] Zhang M., Dai D., Li X., Zhang H., Ma L. (2016). Advances on the study of flowering time trait in hybrid rice. J. Nucl. Agric. Sci..

[6] Meng S., Xu P., Zhang Y., Wang H., Cao L., Cheng S., Shen X. (2018). CRISPR/Cas9-mediated editing of *GS3* to improve flowering time in japonica rice. Chin. J. Rice Sci..

[7] Quan D., Meng W., Jin C., Zhou G. (2016). The effect of flowering habits of CMS Lines in hainan ecological conditions. Crops.

[8] Zhou J., Zhang Y., Zhu D., Lin X., Xiang J., Chen H., Hu S. (2014). Influence of flowering characteristics on spikelet fertility under high temperature. Chin. J. Rice Sci..

[9] Zhang W., Wang Y., Zhu D., Chen H., Xiang J., Zhang Y., Zhang Y. (2019). Effect of increasing night temperature on floret opening and grain setting of rice. Chin. J. Agrometeorol..

[10] Ma Z., Zhan Z., Cheng X., Gao J., He G., Liu D., Xu H., Xu Z. (2011). Flowering time in filial generations of cross between indica and japonica rice and iIts response to external environment. Hybrid Rice.

[11] Gu Y., Wang Z., Gao Y. (1993). An investigation on the effects of environmental factors on the opening and closure of florets in rice. Acta Phytophysiol. Sin..

[12] Kobayasi K. (2012). Effects of solar radiation on fertility and the flower opening time in rice under heat stress conditions. Sol. Radiat..

[13] Ma Z., Zhan Z., Xu H., Xu Z., Mao T., Zhu C., Guo Y. (2011). QTL analysis on flowering time in filial generations of cross between indica and japonica rice. Plant Physiol. J..

[14] Hu X., Chen G., Zhang R., Xu M., Zhao L., Tang H., Ni J., Zhou M. (2023). Multi-Year QTL Mapping and RNA-seq Reveal Candidate Genes for Early Floret-Opening Time in Japonica Rice. Agriculture.

[15] Xu P., Wu T., Ali A., Zhang H., Liao Y., Chen X., Tian Y., Wang W., Fu X., Li Y. (2022). EARLY MORNING FLOWERING1 (EMF1) regulates the floret opening time by mediating lodicule cell wall formation in rice. Plant Biotechnol. J..

[16] Wang M., Zhu X., Peng G., Liu M., Zhang S., Chen M., Liao S., Wei X., Xu P., Tan X. (2022). Methylesterification of cell-wall pectin controls the diurnal flower-opening times in rice. Mol. Plant.

[17] Gou Y., Heng Y., Ding W., Xu C., Tan Q., Li Y., Fang Y., Li X., Zhou D., Zhu X. (2024). Natural variation in OsMYB8 confers diurnal floret opening time divergence between indica and japonica subspecies. Nat. Commun..

[18] Li X., Wang Y., Duan E., Qi Q., Zhou K., Lin Q., Wang D., Wang Y., Long W., Zhao Z. (2018). OPEN GLUME1: A key enzyme reducing the precursor of JA, participates in carbohydrate transport of lodicules during anthesis in rice. Plant Cell Rep..

[19] Zhai X.-w., Kai W.-b., Huang Y.-m., Chen J.-y., Zeng X.-c. (2023). *OsNCED3* and *OsPYL1* promote the closure of rice florets by regulating sugar transporters through endogenous abscisic acid. J. Integr. Agric..

[20] Xiao Y., Chen Y., Charnikhova T., Mulder P.P.J., Ouwerkerk P.B.F. (2014). OsJAR1 is required for JA-regulated floret opening and anther dehiscence in rice. Plant Mol. Biol..

[21] Wang M., Chen M., Huang Z., Zhou H., Liu Z. (2023). Advances on the Study of Diurnal Flower-Opening Times of Rice. Int. J. Mol. Sci..

[22] Huang J., He Y., Zeng X., Xiang M., Fu Y. (2015). Changes of JA Levels in floral organs and expression analysis of JA signaling genes in lodicules before floret opening in rice. Sci. Agric. Sin..

[23] Li L., Zhengshan Z., Ke Q., Chan X., Ying H., Hanlai Z., Xie Z., Michael R., Changxi Y. (2017). Jasmonic acid deficiency leads to scattered floret opening time in cytoplasmic male sterile rice Zhenshan 97A. J. Exp. Bot..

[24] Yan Z., Deng R., Zhang H., Li J., Zhu S. (2022). Transcriptome analysis of floret opening and closure both Indica and Japonica rice. 3 Biotech.

[25] Fu Y., Xiang M., Jiang H., He Y., Zeng X. (2016). Transcriptome profiling of lodicules before floret opening in *Oryza sativa* L.. Sci. Agric. Sin..

[26] Zeng X.C., Zhou X., Zhang W., Murofushi N., Kitahara T., Kamuro Y. (1999). Opening of Rice Floret in Rapid Response to Methyl Jasmonate. J. Plant Growth Regul..

[27] Yan Z., Xu H., Ma Z., Gao D., Xu Z. (2014). Differential response of floret opening to exo-methyl jasmonate between subsp. indica and subsp. japonica in rice. Sci. Agric. Sin..

[28] Yang Y., Xia Y., Yan Z., Wang L., Yan B., Wang J., Wang W., Luan T., Xu H. (2021). Effect of spraying methyl jasmonate during heading date on floret opening time and plant type of japonica rice from different ecological areas. Chin. J. Rice Sci..

[29] Xia Y., Du Z., Yang Y., Gong Y., Yan Z., Xu H. (2019). Effects of epi-brassinolide treatments on floret opening time of indica and japonica rice. Crops.

[30] Yan Z., Xu H., Gong Y., Xia Y., Ma Z., Xu Z. (2015). Difference of floret opening time between subsp. indica and subsp. japonica rice in response to ethephon. J. Shenyang Agric. Univ..

[31] Zeng X. (2000). Induction effect of jasmonates on floret opening in *Oryza sativa* L., *Sorghum bicolar* (L.) Moench and *Dactylis glomerata* L.. Ph.D. Dissertation.

[32] He Y., Zhang F. (2023). Study of regulating effect of auxin on floret opening in rice. Acta Agron. Sin..

[33] Li H. (2009). Plant Microscopy Techniques.

[34] Kim D., Paggi J.M., Park C., Bennett C., Salzberg S.L. (2019). Graph-based genome alignment and genotyping with HISAT2 and HISAT-genotype. Nat. Biotehnol..

[35] Martin M. (2011). Cutadapt removes adapter sequences from high-throughput sequencing reads. EMBnet. J..

[36] Pan X., Welti R., Wang X. (2010). Quantitative analysis of major plant hormones in crude plant extracts by high-performance liquid chromatography-mass spectrometry. Nat. Protoc..

[37] Wang X. (2006). Principles and Techniques of Plant Physiology and Biochemistry Experiments.

[38] Ding W., Gou Y., Li Y., Li J., Fang Y., Liu X., Zhu X., Ye R., Heng Y., Wang H. (2024). A jasmonate-mediated regulatory network modulates diurnal floret opening time in rice. New Phytol..

[39] Wang Z., Zhou Y., Ren X.-Y., Wei K., Fan X.-L., Huang L.-C., Zhao D.-S., Zhang L., Zhang C.-Q., Liu Q.-Q. (2023). Co-Overexpression of Two Key Source Genes, OsBMY4 and OsISA3, Improves Multiple Key Traits of Rice Seeds. J. Agric. Food Chem..

[40] Wang Z., Gu Y., Gao Y. (1994). Studies on the mechanism of the anthesis of rice V. comparison of lodicule and filament structure between sterile line and fertile line. Acta Agron. Sin..

[41] Zhang J., Zhou S., He F., Liu L., Zhang Y., He J., Du X. (2023). Expression pattern of the rice α-amylase genes related with the process of floret opening. Sci. Agric. Sin..

