# Supplementary material for: Revealing Physiological Basis for Floret Opening Difference Between Indica and Japonica Rice: Based on Floral Structure, Transcriptome, and Endogenous Floret Opening Regulator
Source: Genes (Basel). 2024 Oct 30;15(11):1396. doi: 10.3390/genes15111396 (PMC11593404; doi:10.3390/genes15111396)
Supplement: Supplementary file 1 [file genes-15-01396-s001.zip › supplement-Figure.docx]

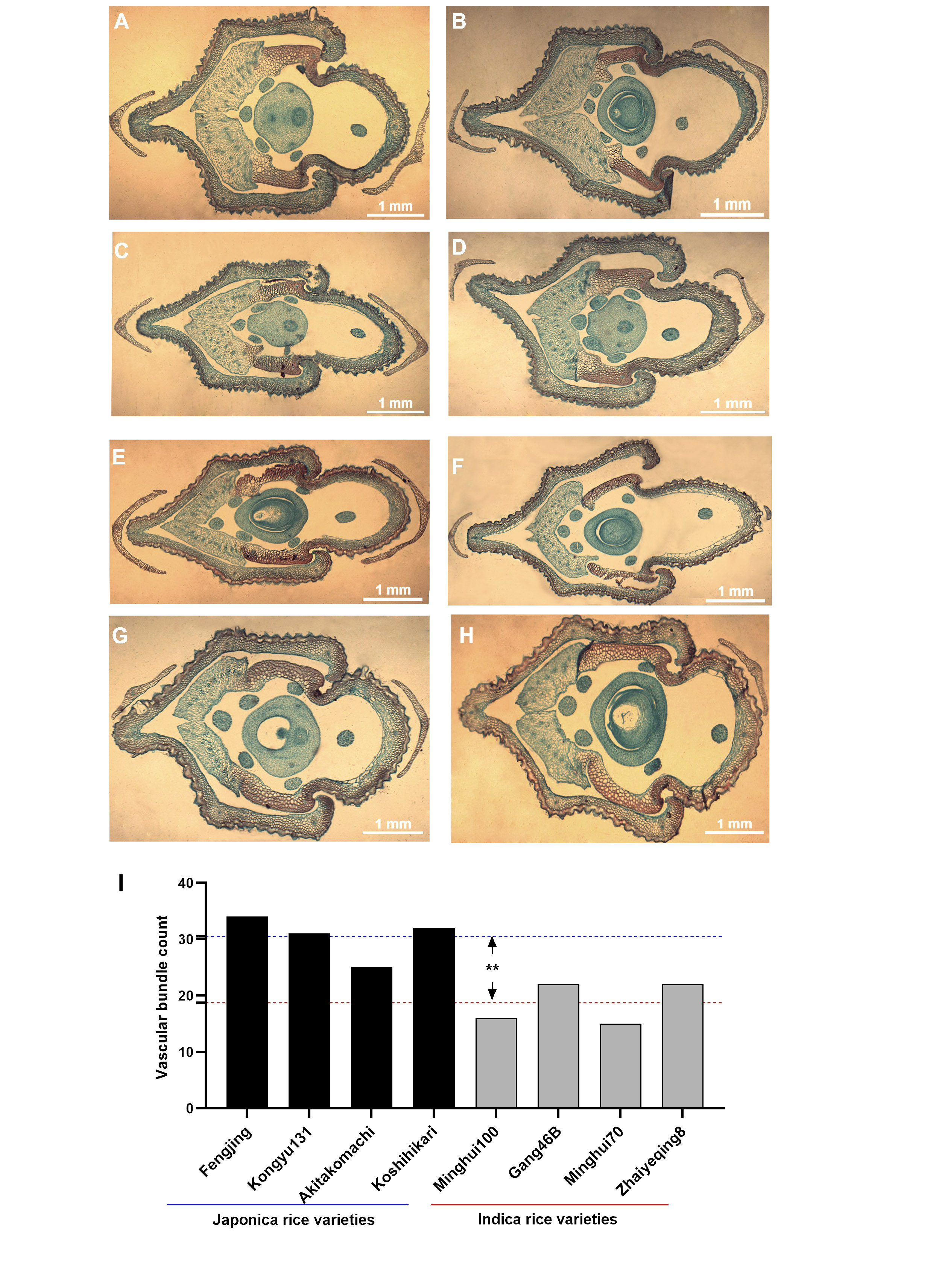


**Figure S1.** Observation of vascular bundles of the lodicule in indica and japonica rice. **(A**–**D)** Japonica rice varieties, respectively: Fengjing, Kongyu131, Akitakomachi, Koshihikari. **(E**–**G)** Indicate rice varieties, respectively: Zhaiyeqing8, Gang46B, Minghui100, Minghui70. **(I)** Comparison of vascular bundle count of lodicule between indica and japonica rice varieties. The blue and red dashed lines show the average vascular bundle numbers in japonica and indica rice lodicules. “**” mean extremely significant at the levels of 0.01.


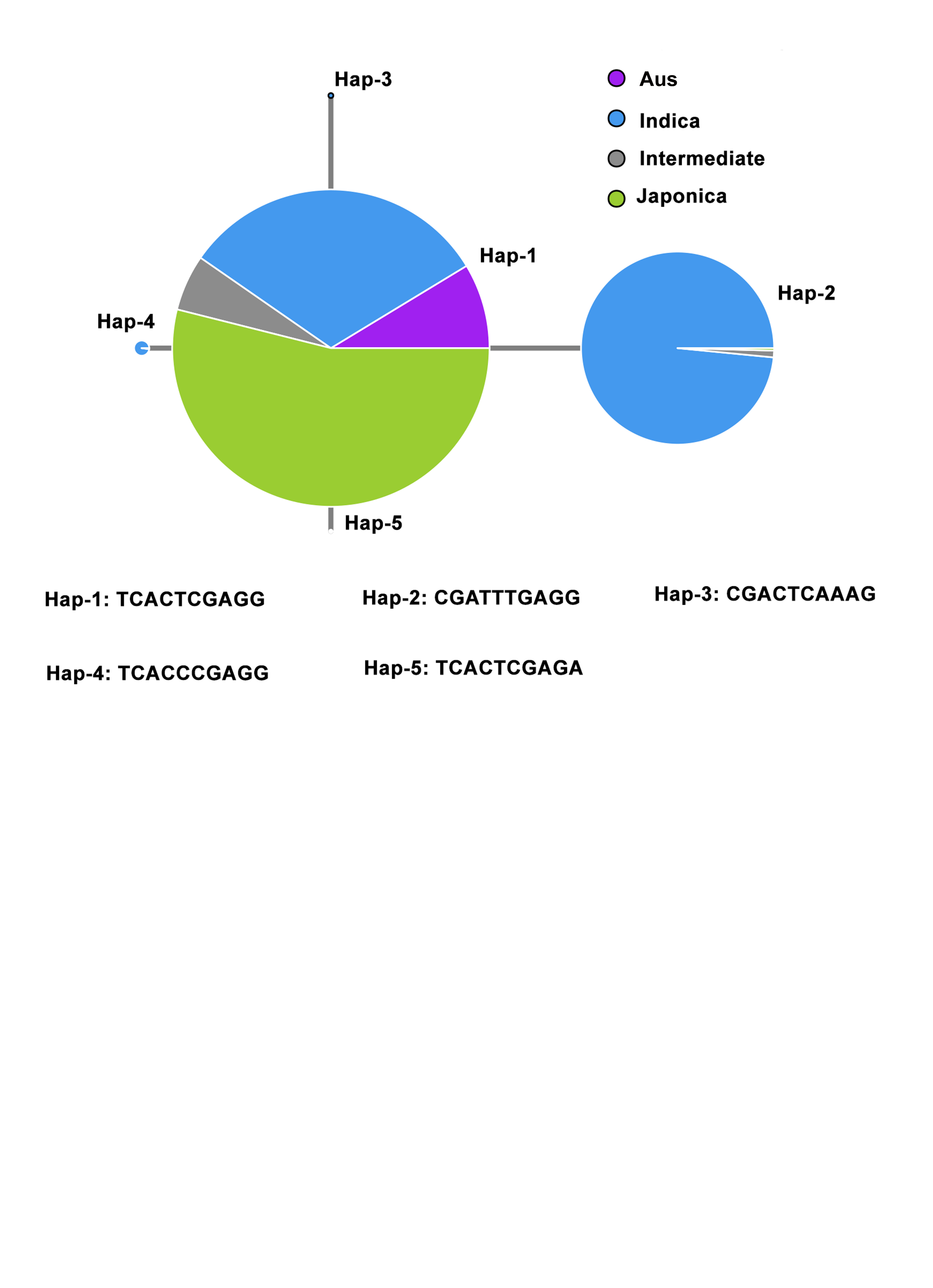


**Figure S2.** Haplotype analysis of *OsAOS1*.


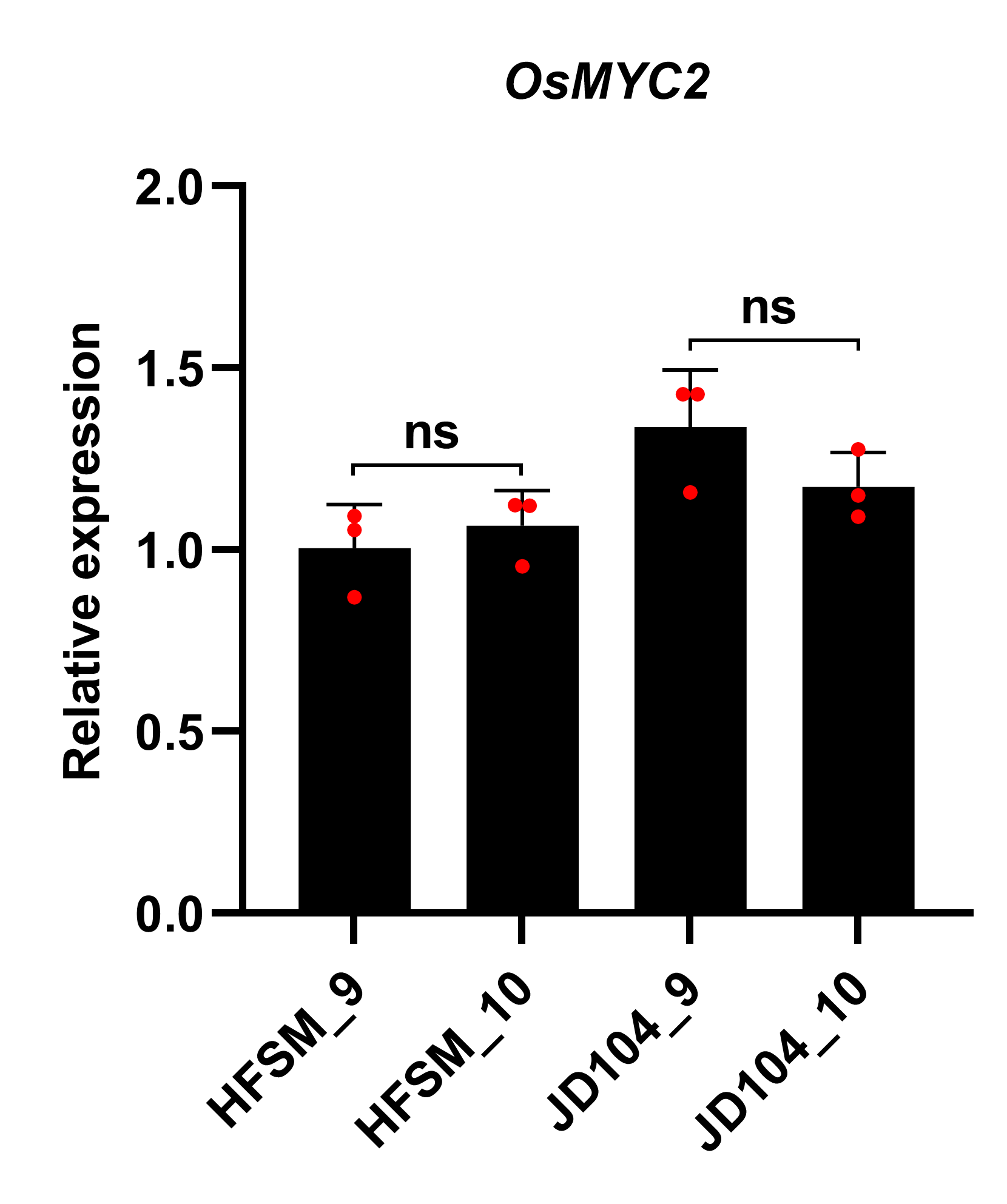


**Figure S3.** Detection of *OsMYC2* expression by RT-PCR. Each red dot represents one replicate.


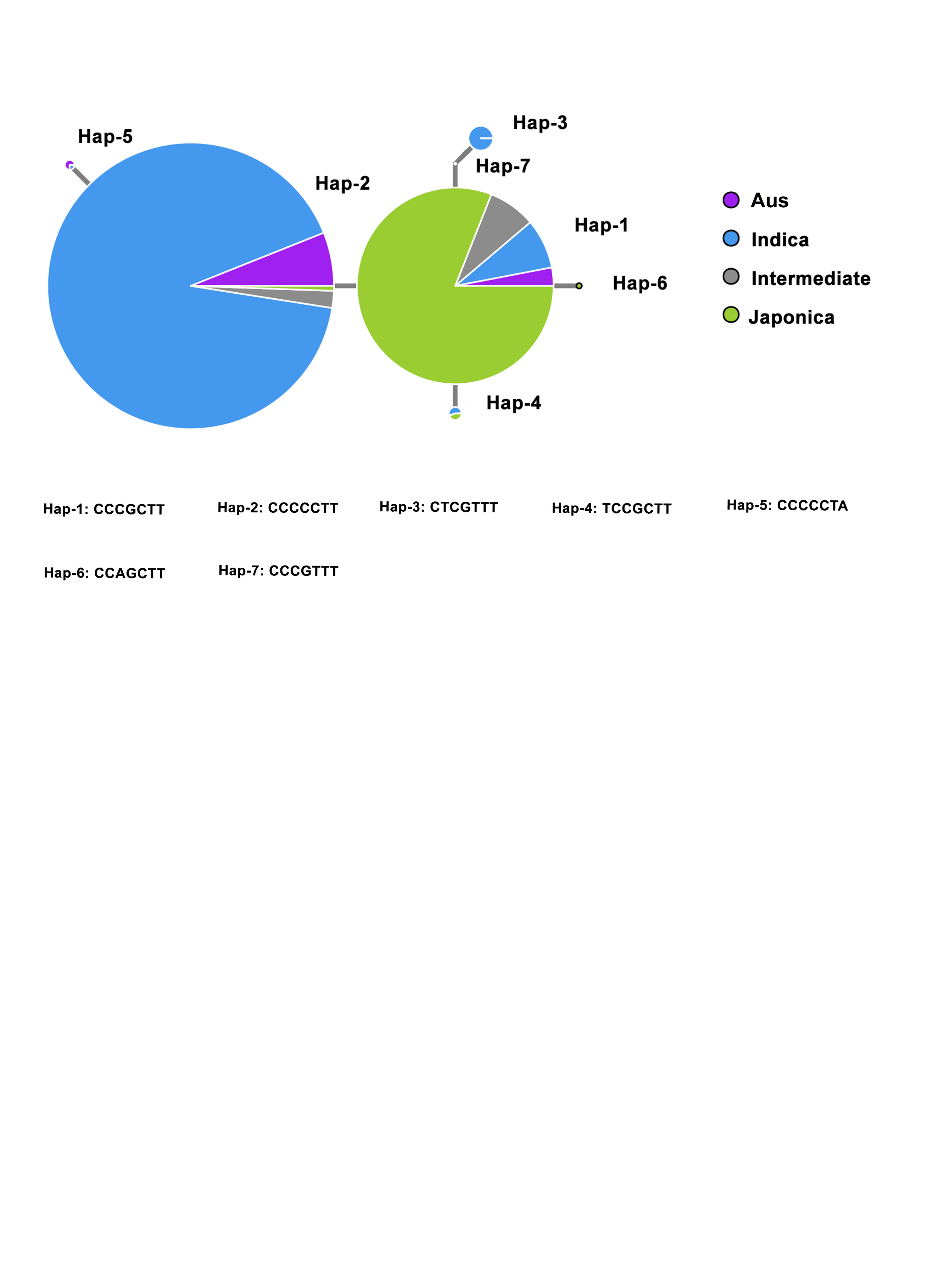


**Figure S4.** Haplotype analysis of *OsMYC2*.
