# Supplementary material for: Revealing Physiological Basis for Floret Opening Difference Between Indica and Japonica Rice: Based on Floral Structure, Transcriptome, and Endogenous Floret Opening Regulator
Source: Genes (Basel). 2024 Oct 30;15(11):1396. doi: 10.3390/genes15111396 (PMC11593404; doi:10.3390/genes15111396)
Supplement: Supplementary file 1 [file genes-15-01396-s001.zip › Table S1.docx]

**Table S1.** Investigation of floret opening time.

| **Cultivar name** | **Variety name abbreviation** | **Subsp. *Indica*/ Subsp. *Japonica*** | **Floret opening time (min) _August 5** | **Floret opening time (min) _August 6** | **Floret opening time (min) _August 12** | **Mean (min)** |
| --- | --- | --- | --- | --- | --- | --- |
| Efengsimiao | EFSM | Subsp. *Indica* | 135 | 133 | 125 | 131 |
| Chenghui727 | CH727 | Subsp. *Indica* | 135 | 133 | 125 | 131 |
| Guihui963 | GH963 | Subsp. *Indica* | 135 | 133 | 125 | 131 |
| Qianxianghui875 | QXH875 | Subsp. *Indica* | 135 | 133 | 134 | 134 |
| Xiangyaxiangzhan | XYXZ | Subsp. *Indica* | 140 | 150 | 134 | 141.33 |
| Jinmazhan | JMZ | Subsp. *Indica* | 135 | 135 | 134 | 134.67 |
| ChengfengB | CFB | Subsp. *Indica* | 147 | 150 | 134 | 143.67 |
| IR8 | IR8 | Subsp. *Indica* | 145 | 135 | 130 | 136.67 |
| Hengfengsimiao | HFSM | Subsp. *Indica* | 135 | 130 | 134 | 133 |
| Jindao104 | JD104 | Subsp. *Japonica* | 170 | 180 | 190 | 180 |
| Shennong9816 | SN9816 | Subsp. *Japonica* | 188 | 191 | 170 | 183 |
| Jinxianggeng | JXG | Subsp*. Japonica* | 150 | 191 | 170 | 170.33 |
| Hugeng1 | HG1 | Subsp. *Japonica* | 215 | 196 | 170 | 193.67 |
| Jigeng88 | JG88 | Subsp. *Japonica* | 175 | 168 | 170 | 171 |
| Jigeng81 | JG81 | Subsp. *Japonica* | 180 | 165 | 160 | 168.33 |
| Chugeng7 | CG7 | Subsp. *Japonica* | 178 | 167 | 165 | 170 |
| Bidao08 | BD08 | Subsp. *Japonica* | 183 | 175 | 165 | 174.33 |
| Yonugeng22 | YG22 | Subsp. *Japonica* | 180 | 167 | 161 | 169.33 |
| JinyuanE28 | JYE28 | Subsp. *Japonica* | 185 | 167 | 165 | 172.33 |
