# Supplementary material for: Revealing Physiological Basis for Floret Opening Difference Between Indica and Japonica Rice: Based on Floral Structure, Transcriptome, and Endogenous Floret Opening Regulator
Source: Genes (Basel). 2024 Oct 30;15(11):1396. doi: 10.3390/genes15111396 (PMC11593404; doi:10.3390/genes15111396)
Supplement: Supplementary file 1 [file genes-15-01396-s001.zip › Table S2.docx]

**Table S2.** Weather records.

| **Data** | **Maximum temperature (°C)** | **Minimum temperature (°C)** | **Weather conditions** |
| --- | --- | --- | --- |
| 5 August 2024 | 30 | 21 | Sunny |
| 6 August 2024 | 31 | 22 | Sunny |
| 7 August 2024 | 30 | 22 | cloudy |
| 8 August 2024 | 30 | 23 | cloudy |
| 9 August 2024 | 30 | 22 | cloudy |
| 10 August 2024 | 27 | 21 | Rain |
| 11 August 2024 | 26 | 21 | Rain |
| 12 August 2024 | 30 | 20 | Sunny |
