# Supplementary material for: Revealing Physiological Basis for Floret Opening Difference Between Indica and Japonica Rice: Based on Floral Structure, Transcriptome, and Endogenous Floret Opening Regulator
Source: Genes (Basel). 2024 Oct 30;15(11):1396. doi: 10.3390/genes15111396 (PMC11593404; doi:10.3390/genes15111396)
Supplement: Supplementary file 1 [file genes-15-01396-s001.zip › Table S3.docx]

**Table S3.** The measurements of the floral organs.

| **Cultivar name** | **Variety name abbreviation** | **Subsp. *Indica*/Subsp. *Japonica*** | **Glume thickness(μm)** | **Lodicule thickness (μm)** | **Lodicule area (μm^2^)** | **Coupling-lodicule length (μm)** | **Lodicule-lemma gap (μm)** | **Vascular bundle count** |
| --- | --- | --- | --- | --- | --- | --- | --- | --- |
| Efengsimiao | EFSM | Subsp. *Indica* | 159.02 | 183.10 | 120,878.50 | 302.90 | 38.50 | 18 |
| Chenghui727 | CH727 | Subsp. *Indica* | 47.18 | 221.95 | 188,216.90 | 426.60 | 47.18 | 18 |
| Guihui963 | GH963 | Subsp. *Indica* | 133.65 | 211.90 | 174,278.20 | 379.30 | 32.10 | 16 |
| Qianxianghui875 | QXH875 | Subsp. *Indica* | 158.12 | 269.95 | 227,512.70 | 472.75 | 42.58 | 22 |
| Xiangyaxiangzhan | XYXZ | Subsp. *Indica* | 141.35 | 220.60 | 169,720.80 | 365.70 | 32.18 | 21 |
| Jinmazhan | JMZ | Subsp. *Indica* | 161.07 | 180.40 | 129,225.70 | 331.80 | 40.27 | 23 |
| ChengfengB | CFB | Subsp. *Indica* | 167.40 | 172.50 | 128,860.70 | 359.20 | 49.88 | 19 |
| IR8 | IR8 | Subsp. *Indica* | 174.55 | 193.90 | 130,405.00 | 448.10 | 76.20 | 24 |
| Hengfengsimiao | HFSM | Subsp. *Indica* | 158.58 | 253.80 | 258,298.30 | 418.85 | 59.88 | 40 |
| Jindao104 | JD104 | Subsp. *Japonica* | 205.22 | 213.50 | 109,650.50 | 352.20 | 52.17 | 29 |
| Shennong9816 | SN9816 | Subsp. *Japonica* | 145.02 | 215.30 | 222,213.30 | 407.05 | 42.53 | 30 |
| Jinxianggeng | JXG | Subsp. *Japonica* | 176.05 | 195.25 | 202,246.50 | 314.80 | 67.07 | 36 |
| Hugeng1 | HG1 | Subsp. *Japonica* | 159.53 | 248.60 | 242,890.90 | 324.10 | 90.62 | 34 |
| Jigeng88 | JG88 | Subsp. *Japonica* | 186.55 | 264.90 | 235,033.10 | 413.30 | 41.15 | 39 |
| Jigeng81 | JG81 | Subsp. *Japonica* | 147.40 | 180.55 | 134,855.20 | 365.35 | 44.12 | 28 |
| Chugeng7 | CG7 | Subsp. *Japonica* | 154.08 | 235.05 | 172,683.20 | 379.70 | 85.87 | 38 |
| Bidao08 | BD08 | Subsp. *Japonica* | 166.73 | 183.25 | 145,394.00 | 367.90 | 78.82 | 32 |
| Yonugeng22 | YG22 | Subsp. *Japonica* | 186.95 | 212.40 | 184,082.50 | 376.40 | 64.47 | 38 |
| JinyuanE28 | JYE28 | Subsp. *Japonica* | 155.62 | 214.65 | 203,301.00 | 396.20 | 72.32 | 38 |
