# Supplementary material for: Revealing Physiological Basis for Floret Opening Difference Between Indica and Japonica Rice: Based on Floral Structure, Transcriptome, and Endogenous Floret Opening Regulator
Source: Genes (Basel). 2024 Oct 30;15(11):1396. doi: 10.3390/genes15111396 (PMC11593404; doi:10.3390/genes15111396)
Supplement: Supplementary file 1 [file genes-15-01396-s001.zip › Table S4.docx]

**Table S4.** RNA integrity number (RIN) and sequencing data.

| **Sample** | **Sampling time** | **RIN** | **Raw Data (Reads)** | **Base** | **Valid Data (Reads) %** | **Base** | **Valid Ratio (reads)** | **Q20%** | **Q30%** |
| --- | --- | --- | --- | --- | --- | --- | --- | --- | --- |
|  |  |  |  |  |  |  |  |  |  |
| HFSM_9_1 | 9:00 a.m. | 8.62 | 37485404 | 5.62G | 36574524 | 5.49G | 97.57 | 99.97 | 97.47 |
| HFSM_9_2 | 9:00 a.m. | 8.58 | 52183322 | 7.83G | 50920336 | 7.64G | 97.58 | 99.97 | 97.47 |
| HFSM_9_3 | 9:00 a.m. | 8.47 | 36380150 | 5.46G | 35532414 | 5.33G | 97.67 | 99.97 | 97.33 |
| HFSM_10_1 | 10:00 a.m. | 8.38 | 38903676 | 5.84G | 38015722 | 5.70G | 97.72 | 99.96 | 97.21 |
| HFSM_10_2 | 10:00 a.m. | 8.55 | 43331224 | 6.50G | 42290850 | 6.34G | 97.6 | 99.96 | 97.21 |
| HFSM_10_3 | 10:00 a.m. | 8.5 | 37945776 | 5.69G | 37045952 | 5.56G | 97.63 | 99.96 | 97.16 |
| JD104_9_1 | 9:00 a.m. | 8.31 | 44738884 | 6.71G | 43579080 | 6.54G | 97.41 | 99.97 | 97.39 |
| JD104_9_2 | 9:00 a.m. | 8.23 | 39421492 | 5.91G | 38437700 | 5.77G | 97.5 | 99.97 | 97.33 |
| JD104_9_3 | 9:00 a.m. | 7.76 | 36823256 | 5.52G | 35687652 | 5.35G | 96.92 | 99.77 | 96.9 |
| JD104_10_1 | 10:00 a.m. | 8.23 | 51280612 | 7.69G | 49923884 | 7.49G | 97.35 | 99.97 | 97.55 |
| JD104_10_2 | 10:00 a.m. | 8.18 | 41941752 | 6.29G | 40850478 | 6.13G | 97.4 | 99.97 | 97.26 |
| JD104_10_3 | 10:00 a.m. | 8.21 | 37649008 | 5.65G | 35909478 | 5.39G | 95.38 | 99.17 | 96.06 |
