# Supplementary material for: Revealing Physiological Basis for Floret Opening Difference Between Indica and Japonica Rice: Based on Floral Structure, Transcriptome, and Endogenous Floret Opening Regulator
Source: Genes (Basel). 2024 Oct 30;15(11):1396. doi: 10.3390/genes15111396 (PMC11593404; doi:10.3390/genes15111396)
Supplement: Supplementary file 1 [file genes-15-01396-s001.zip › Table S5.docx]

**Table S5.** Primer’s information.

| **Gene ID** | **Gene** | **Primer** | **Primer sequence** |
| --- | --- | --- | --- |
| *Os03g0767000* | *AOS1* | *OsAOS1*-QF | TTCTACCTCCTCTCCCACCG |
|  |  | *OsAOS1*-QR | CGAGGTCGTTCTCCATGAGG |
| *Os09g0469400* | *OsISA3* | *OsISA3*-QF | CCCCACCACCACACCATAAA |
|  |  | *OsISA3*-QR | TGGTCCTGTAAATGGCACCC |
| *Os10g0575000* | *OsMYC2* | *OsMYC2*-QF | CCCCCATGGACATGAAGGAC |
|  |  | *OsMYC2*-QR | CCGTTCTCGAAGTGCTGGAT |
|  | *GAPDH* (Internal reference) | *GAPDH*-QF | AAGCCAGCATCCTATGATCAGATT |
|  |  | *GAPDH*-QR | CGTAACCCAGAATACCCTTGAGTTT |
